# Data set for cloning and characterization of heterologous transporters in *Saccharomyces cerevisiae* and identification of important amino acids for xylose utilization

**DOI:** 10.1016/j.dib.2015.05.005

**Published:** 2015-05-19

**Authors:** Chengqiang Wang, Xiaoming Bao, Yanwei Li, Chunlei Jiao, Jin Hou, Qingzhu Zhang, Weixin Zhang, Weifeng Liu, Yu Shen

**Affiliations:** a The State Key Laboratory of Microbial Technology, Shandong University, Shan Da Nan Road27, Jinan 250100, PR China; b College of Life Sciences, Shandong Agricultural University/Shandong Key Laboratory of Agricultural Microbiology, Daizong Street 61, Taian 271018, PR China; c Environment Research Institute, Shandong University, Shan Da Nan Road 27, Jinan 250100, PR China

## Abstract

The efficient uptake is important for the xylose utilization by *Saccharomyces cerevisiae*. A heterogenous transporter Mgt05196p was cloned from *Meyerozyma guilliermondii* and expressed in *Saccharomyces cerevisiae*[1]. This data article contains the transport characteristics of Mgt05196p in *S. cerevisiae.* The fluorescence of fusion protein Mgt05196p-GFP expressing strain was located on the cell surface demonstrated that the heterogenous transporter Mgt05196p was targeted to the plasma membrane of *S. cerevisiae*. The expressing of Mgt05196p in the hxt null *S. cerevisiae* endowed the strain with the glucose and d-xylose absorption capacity, as well as expressing the native d-xylose transporter Gal2p. The transmembrane domains of Mgt05196p were predicted and compared with the XylEp, whose crystal structure was revealed. And then, the homologous modeling of Mgt05196p was built basing on the XylEp to find out the crucial amino acid residues for sugars binding and transport.

**Specifications table**

**Table:** 

Subject area	Biology
More specific subject area	Membrane transporter
Type of data	Image, figure, table
How data was acquired	The epifluorescence and phase-contrast images were acquired using a Nikon ECLIPSE 80i system. The metabolic products analysis was acquired by HPLC using a Prominence LC-20A. The transmembrane domain prediction was acquired by using software HMMTOP. The homologous modeling was acquired using the software Discovery Studio.
Data format	Raw and analyzed
Experimental factors	No pretreatment
Experimental features	Fluorescence microscopy of protein; batch fermentation; HPLC; transmembrane domain prediction, homologous modeling.
Data source location	Not applicable
Data accessibility	Data is supplied with this article

## 1 Value of the data

• The fluorescence microscopic image demonstrated that the heterogenous transporter Mgt05196p was targeted to the plasma membrane of *S. cerevisiae*.
• The fermentation result revealed that the Mgt05196p acted as a transporter of glucose and d-xylose in *S. cerevisiae.*
• The homologous modeling of Mgt05196p suggested the amino acid residues that might be crucial for xylose binding and transport.
• The intracellular d-xylose accumulation assays indicated the crucial amino acid residues for xylose transport.

## 2 Data, experimental design, materials and methods

### 2.1 Fluorescence microscopy of transporter Mgt05196p

**Fig. 1:**
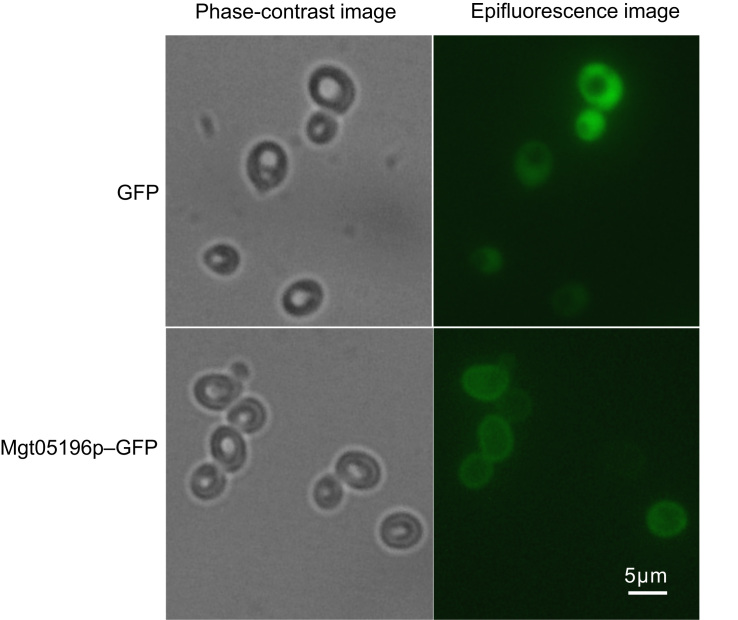
The fluorescence image of strain expressing fusion protein Mgt05196p-GFP and GFP. The Mgt05196p gene was fused with the GFP gene at the 3′ end and expressed in the hxt null strain EBY.VW4000. Unlike the GFP expressing strain, in which the fluorescence was dispersive, the fluorescence of the fusion protein Mgt05196p-GFP expressing strain was localized to the cell surface. Strains: the hxt null cells EBY.VW4000 expressed the GFP (reference) and GFP fusion protein Mgt05196p-GFP.

The green fluorescent protein was fused to the 3′ end of Mgt05196p to verify its location in *S. cerevisiae*. The fragment of *MGT05196* gene without a stop codon was inserted in front of the encoding DNA sequence of yEGFP3 in plasmid pJFE3-yEGFP3 [2], resulting pJFE3-MGT05196-yEGFP3. The plasmids pJFE3-yEGFP3 and pJFE3-MGT05196-yEGFP3 were transformed into the hxt null strain EBY.VW4000, which lacks all of the 18 native hexose transporters [3]. Single colonies of the constructed strains were firstly cultured for 10 h, harvested, and then resuspended in 50 mmol L^−1^ PBS buffer (pH 6.5). A Nikon ECLIPSE 80i system equipped with plan Apochromats 40× objective (NA=0.95) and 60× oil objective (NA=1.40) was used to take the epifluorescence and phase-contrast images as reported [4]. Different with the GFP expressing strain, in which the fluorescence was dispersive, the fluorescence of fusing protein Mgt05196p-GFP expressing strain was focused on the cell surface (Fig. 1), demonstrated that heterogenous transporter Mgt05196p was accurately targeted to the plasma membrane.

### 2.2 Batch fermentation and products analysis

**Fig. 2:**
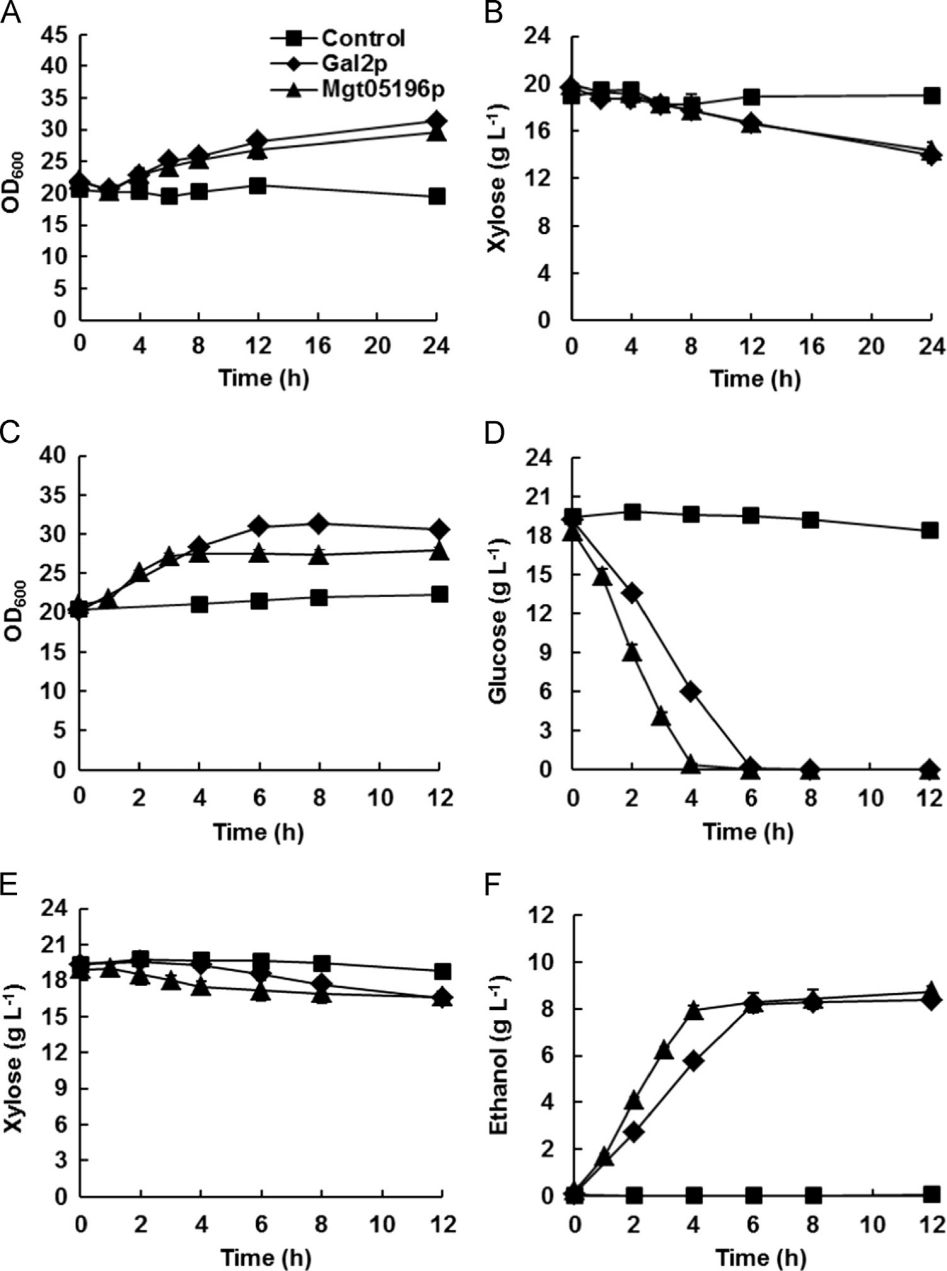
The fermentation characteristics of the strains. The strain growth (A) and D-xylose consumption (B) in d-xylose aerobic fermentation. The strain growth (C), glucose consumption (D), d-xylose consumption (E), and ethanol production (F) in d-xylose and glucose oxygen-limited cofermentation. The 12 h precultured cells were collected, washed, and inoculated into 40 mL of SD medium plus 20 g L^−1^ D-xylose or 20 g L^−1^ D-xylose and 20 g L^−1^ glucose, with an initial OD_600_ of 20 (4.8 g DCW L^−1^). The error bars represent the standard deviation of the biological triplicates. All of the strains were derived from the hxt null strain BSW4EYX, whose genome integrated the set of genes of XR, XDH, and XK. The control was strain BSW4PPX, which contained the empty plasmid for transporter expression. The other strains are represented by the transporters they expressed.

The transporters Mgt05196p and native Gal2p were expressed in the hxt null strain EBY.VW4000 [3] expressing d-xylose metabolic pathway genes to study the function of Mgt05196p to d-xylose transport and metabolism. The pre-cultured cells were collected, washed and inoculated in 100 mL flasks with 40 mL of SD medium, which containing 1.7 g L^−1^ yeast nitrogen base (YNB, Sangon, China), 5 g L^−1^ ammonium sulfate (Sangon, China), CSM-URA (MP Biomedicals, Solon, OH) and sugars. Then they were cultured at 30 °C, 200 r min^−1^. The aerobic and oxygen-limited conditions were maintained using cotton plugs and rubber stoppers on the shake flask, respectively. The concentration of d-xylose, glucose, and ethanol were determined by HPLC using a Prominence LC-20A (Shimadzu, Japan) equipped with the refractive index detector RID-10A (Shimadzu, Japan) and the Aminex HPX-87H ion exchange column (Bio-Rad, Hercules, USA), as formerly reported [5]. The strains with the transporters Mgt05196p and Gal2p grew on d-xylose and glucose (Fig. 2), while the control strain with the empty plasmid did not grow on either d-xylose or glucose. During aerobic d-xylose fermentation, the maximum specific d-xylose consumption rate of the strains with Mgt05196p and Gal2p were almost equal (Fig. 2). In the fermentation of medium containing both d-xylose and glucose, the ratio of the specific consumption rate of d-xylose and glucose (X/G) of the Mgt05196p and Gal2p containing cells represents the preference for D-xylose and glucose [6]. In the oxygen-limited fermentation, the X/G of the Mgt05196p expressing strain was ~50% higher than the Gal2p expressing strain, which indicated a higher preference for D-xylose (Fig. 2).

### 2.3 The transmembrane domain prediction

**Fig. 3:**
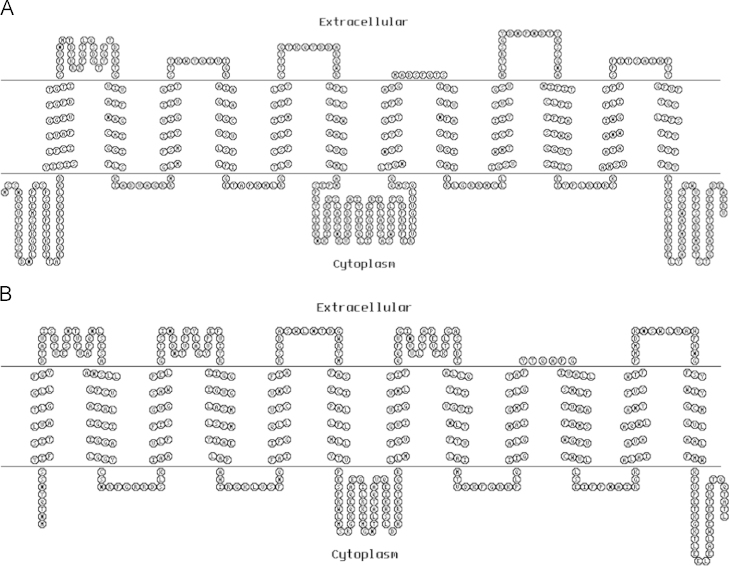
The transmembrane domains prediction of transporters. (A), Protein: Mgt05196p. Length: 560; N-terminus: IN; Number of transmembrane helices: 12; Transmembrane helices: 52-76 107-124 135-152 165-182 193-210 227-244 327-345 354-371 382-400 421-445 456-474 487-506. (B) Protein: XylEp. Length: 491; N-terminus: IN; Number of transmembrane helices: 12; Transmembrane helices: 9-26 53-77 90-107 134-156 169-186 203-220 270-287 314-333 346-363 370-394 407-426 443-460.

The transmembrane domains of Mgt05196p were predicted and compared with the XylEp, whose crystal structure was revealed, using the online tool HMMTOP (http://www.sacs.ucsf.edu/cgi-bin/hmmtop.py) [7]. The result revealed that the Mgt05196p contained 12 similar transmembrane helices, but longer intracellular sequences at the N-terminus and C-terminus (Fig. 3).

### 2.4 Homologous modeling of Mgt05196p and the crucial amino acid residues for D-xylose binding

**Fig. 4:**
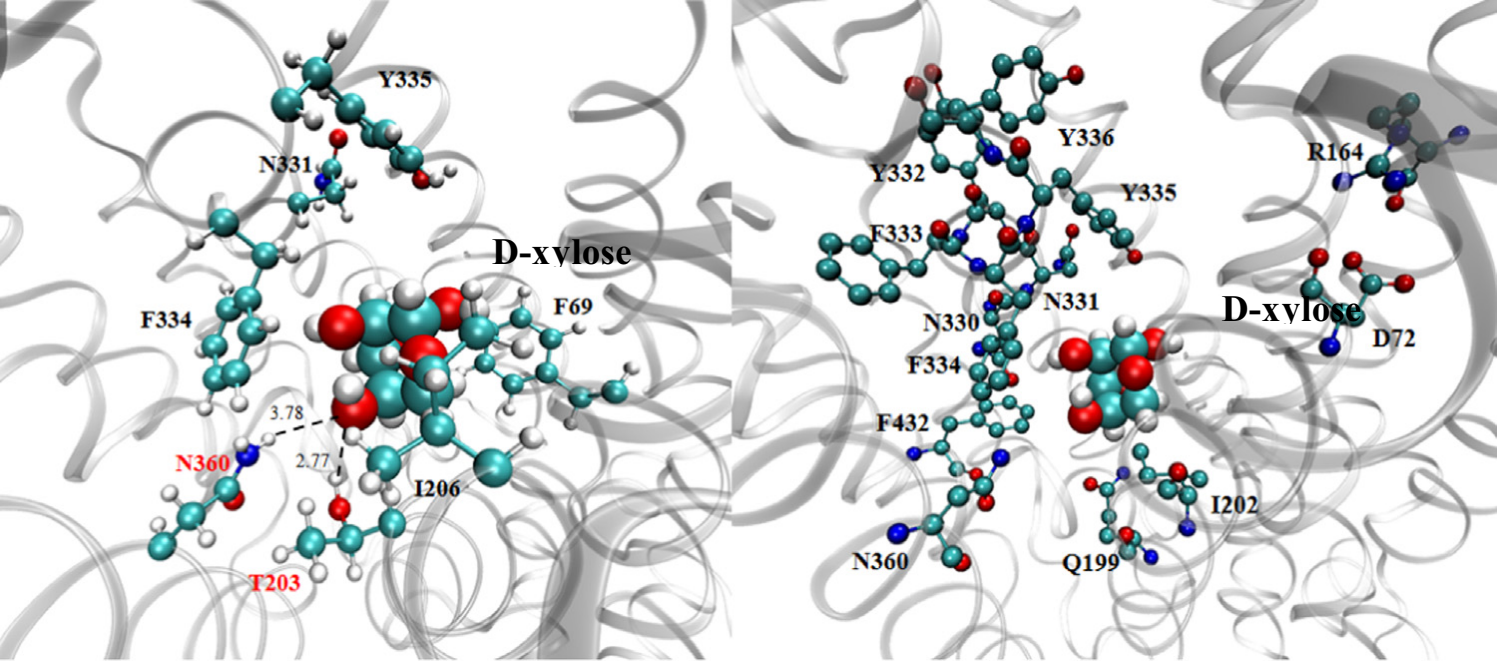
The homology 3 D model of Mgt05196p with D-xylose. The homology model of Mgt05196p was constructed according to the outward-facing and partly occluded structure of XylE using Discovery Studio. The location of D-xylose was determined using core-constrained protein docking and a modified CHARMm-based CDOCKER method. The best position among the 10 calculated positions of D-xylose was chosen by comparing the CDOCKER energies. The middle of the Mgt05196p model showed a colored D-xylose using the space-filling model. The residues of the predicted amino acids are also colorfully presented around D-xylose using the ball-and-stick model.

To study the transport mechanism of Mgt05196p, a 3D structure of Mgt05196p was modeled according to the outward-facing and partly occluded conformation of XylEp [8] using Discovery Studio software (Fig. 4). Basing on the transmembrane domain prediction result of software HMMTOP (Fig. 3), the 49 amino acids in the N-terminal and 39 amino acids in the C-terminal of Mgt05196p were ignored. Ten polar/aromatic amino acid residues F69, Q199, I202, T203, N330, N331, F334, Y335, N360, and F432 are within the distance of 5 Å to D-xylose in the model were selected out. Furthermore; the Q325, Q326, and W465 are corresponding to the residues of XylE for D-xylose binding; the D72 and R164 of Mgt05196p are corresponding to the D27 and R133 of XylE [8–12], or the D22 and R102 of GlcP_se_ [13], which were predicted to be involved in the protonation; and other three amino acid residues Y332, F333, and Y336 in the aromatic residues rich sequence YFFYY, which is involved in the conserved motif of transmembrane section 7 (TMS7) of Mgt05196p were also select out for study. Moreover, basing on the model did not ignore the amino acids in the N-terminal and C-terminal, other 9 amino acid residues F79, K156, Q284, E293, G337, G383, I393, N411, K415, and V440, which are within the distance of 5 Å to D-xylose in the model, were also chosen to verify their role for D-xylose and glucose transport. The 14 sites shown in Fig. 4 had been verified to have effect on D-xylose transport [1] and hold the potential to increase the transport efficiency and specificity. And the position of the selected sites in TMS of Mgt05196p was listed in Table 1.

### 2.5 The effect of mutating the amino acid residues of Mgt05196p on the intracellular D-xylose accumulation.

**Fig. 5:**
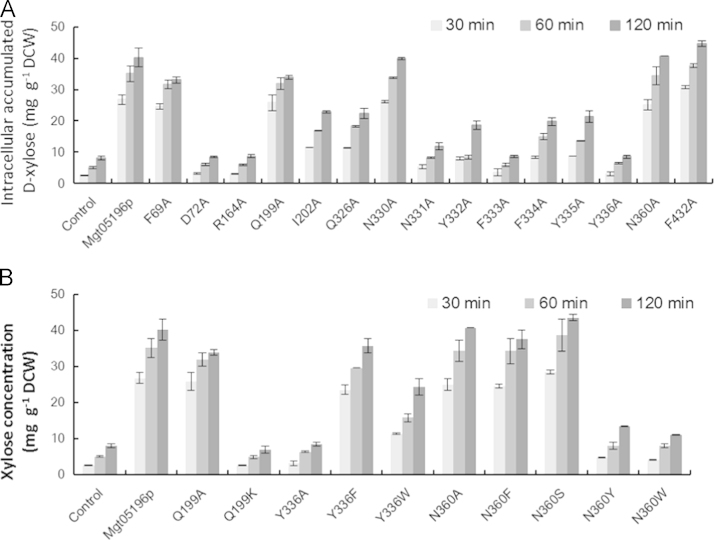
The effect of mutating the amino acid residues of Mgt05196p to alanine (A) or some other residues (B) on the intracellular D-xylose accumulation. The well cultured cells were incubated in SD medium with 20 g L^−1^D-xylose at 30 °C for 30, 60 and 120 min, then the intracellular D-xylose was extract using ddH_2_O [1]. The intracellular D-xylose was defined as the total amount of D-xylose and xylitol per gram in the dried cells. The error bars represent the standard deviation of the biological triplicates. All of the strains were derived from the hxt null strain EBY.VW4000. The control was strain BSW4PP, which contained the empty plasmid. The other strains are represented by the transporters and mutants they expressed.

Natural *S. cerevisiae* cannot grow on D-xylose and L-arabinose, but can convert them into sugar alcohol. Therefore, the pentose transport performance of the transporters was evaluated upon assaying the total intracellular accumulation of sugar and sugar alcohol [14]. Replacing the residues D72, R164, N331, Y332, F333, F334, Y335, and Y336 with alanine significantly reduced the net intracellular D-xylose accumulation, indicating their importance to D-xylose transport (Fig. 5A). Then some of the residues of Mgt05196p were replaced with residues besides alanine. Although changing the Q199 to A did not affect the transporter much, the change of Q199K almost blocked the D-xylose transport. Although the mutant Y336A did not transport D-xylose and glucose, the strain expressing mutant Y336F and Y336W accumulated the D-xylose well. The change of N360 to A, S, and F did not affect the D-xylose accumulation, but mutants N360Y and N360W lost the D-xylose transport capacity (Fig. 5B).

## Conflict of interest

The authors declare that there is no conflict of interest on any work published in this paper.

## Figures and Tables

**Table 1:** The position of the effective D-xylose transport sites in the predicted structure of Mgt05196p.

Transporter or mutant sites	The position in the predicted structure of Mgt05196p
F69	TMS1
D72	TMS1
R164	TMS3–TMS4
Q199	TMS5
I202	TMS5
T203	TMS5
N330	TMS7
N331	TMS7
Y332	TMS7
F333	TMS7
F334	TMS7
Y335	TMS7
Y336	TMS7
N360	TMS8
F432	TMS10
