# Supplementary material for: Data set for cloning and characterization of heterologous transporters in Saccharomyces cerevisiae and identification of important amino acids for xylose utilization
Source: Data Brief. 2015 May 19;4:119–26. doi: 10.1016/j.dib.2015.05.005 (PMC4510445; doi:10.1016/j.dib.2015.05.005)
Supplement: Supplementary file 1 — Supplementary data [file mmc1.zip › Supplementary Fig. 1.doc]

Phase-contrast image	Epiﬂuorescence image

GFP

Mgt05196p–GFP

5μm

**Fig. 1.** The fluorescence image of strain expressing fusion protein Mgt05196p-GFP and GFP. The Mgt05196p gene was fused with the GFP gene at the 3’ end and expressed in the hxt null strain EBY.VW4000. Unlike the GFP expressing strain, in which the fluorescence was dispersive, the fluorescence of the fusion protein Mgt05196p-GFP expressing strain was localized to the cell surface. Strains: The hxt null cells EBY.VW4000 expressed the GFP (reference) and GFP fusion protein Mgt05196p-GFP.
