# Supplementary material for: Data set for cloning and characterization of heterologous transporters in Saccharomyces cerevisiae and identification of important amino acids for xylose utilization
Source: Data Brief. 2015 May 19;4:119–26. doi: 10.1016/j.dib.2015.05.005 (PMC4510445; doi:10.1016/j.dib.2015.05.005)
Supplement: Supplementary file 1 — Supplementary data [file mmc1.zip › Supplementary Fig. 2.doc]

A-xylose

B-xylose

C-xylose

D-xylose

E-xylose

F-xylose

**Fig. 2.** The fermentation characteristics of the strains. The strain growth (A) and D-xylose consumption (B) in D-xylose aerobic fermentation. The strain growth (C), glucose consumption (D), D-xylose consumption (E), and ethanol production (F) in D-xylose and glucose oxygen-limited cofermentation. The 12 h precultured cells were collected, washed, and inoculated into 40 mL of SD medium plus 20 g L^-1^ D-xylose or 20 g L^-1^ D-xylose and 20 g L^-1^ glucose, with an initial OD_600_ of 20 (4.8 g DCW L^-1^). The error bars represent the standard deviation of the biological triplicates. All of the strains were derived from the hxt null strain BSW4EYX, whose genome integrated the set of genes of XR, XDH, and XK. The control was strain BSW4PPX, which contained the empty plasmid for transporter expression. The other strains are represented by the transporters they expressed.
