# Supplementary material for: Data set for cloning and characterization of heterologous transporters in Saccharomyces cerevisiae and identification of important amino acids for xylose utilization
Source: Data Brief. 2015 May 19;4:119–26. doi: 10.1016/j.dib.2015.05.005 (PMC4510445; doi:10.1016/j.dib.2015.05.005)
Supplement: Supplementary file 1 — Supplementary data [file mmc1.zip › Supplementary Fig. 3.doc]

(A)

(B)

**Fig. 3.** The transmembrane domains prediction of transporters. (A), Protein: Mgt05196p. Length: 560; N-terminus: IN; Number of transmembrane helices: 12; Transmembrane helices: 52-76 107-124 135-152 165-182 193-210 227-244 327-345 354-371 382-400 421-445 456-474 487-506. (B) Protein: XylEp. Length: 491; N-terminus: IN; Number of transmembrane helices: 12; Transmembrane helices: 9-26 53-77 90-107 134-156 169-186 203-220 270-287 314-333 346-363 370-394 407-426 443-460.
