# Supplementary material for: Data set for cloning and characterization of heterologous transporters in Saccharomyces cerevisiae and identification of important amino acids for xylose utilization
Source: Data Brief. 2015 May 19;4:119–26. doi: 10.1016/j.dib.2015.05.005 (PMC4510445; doi:10.1016/j.dib.2015.05.005)
Supplement: Supplementary file 1 — Supplementary data [file mmc1.zip › Supplementary Fig. 4.doc]

**D-xylose**

**D-xylose**

**Fig. 4.** The homology 3 D model of Mgt05196p with D-xylose. The homology model of Mgt05196p was constructed according to the outward-facing and partly occluded structure of XylE using Discovery Studio. The location of D-xylose was determined using core-constrained protein docking and a modified CHARMm-based CDOCKER method. The best position among the ten calculated positions of D-xylose was chosen by comparing the CDOCKER energies. The middle of the Mgt05196p model showed a colored D-xylose using the space-filling model. The residues of the predicted amino acids are also colorfully presented around D-xylose using the ball-and-stick model.
