# Supplementary material for: Data set for cloning and characterization of heterologous transporters in Saccharomyces cerevisiae and identification of important amino acids for xylose utilization
Source: Data Brief. 2015 May 19;4:119–26. doi: 10.1016/j.dib.2015.05.005 (PMC4510445; doi:10.1016/j.dib.2015.05.005)
Supplement: Supplementary file 1 — Supplementary data [file mmc1.zip › Supplementary Fig. 5.doc]

**Fig. 5.** The effect of mutating the amino acid residues of Mgt05196p to alanine (A) or some other residues (B) on the intracellular D-xylose accumulation. The well cultured cells were incubated in SD medium with 20 g L^-1^ D-xylose at 30°C for 30, 60 and 120 min, then the intracellular D-xylose was extract using ddH_2_O [1]. The intracellular D-xylose was defined as the total amount of D-xylose and xylitol per gram in the dried cells. The error bars represent the standard deviation of the biological triplicates. All of the strains were derived from the hxt null strain EBY.VW4000. The control was strain BSW4PP, which contained the empty plasmid. The other strains are represented by the transporters and mutants they expressed.
