# Supplementary material for: Data set for cloning and characterization of heterologous transporters in Saccharomyces cerevisiae and identification of important amino acids for xylose utilization
Source: Data Brief. 2015 May 19;4:119–26. doi: 10.1016/j.dib.2015.05.005 (PMC4510445; doi:10.1016/j.dib.2015.05.005)
Supplement: Supplementary file 1 — Supplementary data [file mmc1.zip › Supplementary Table 1.doc]

**Table 1** The position of the effective D-xylose transport sites in the predicted structure of Mgt05196p

| Transporter or mutant sites | The position in the predicted structure of Mgt05196p |
| --- | --- |
| F69 | TMS1 |
| D72 | TMS1 |
| R164 | TMS3-TMS4 |
| Q199 | TMS5 |
| I202 | TMS5 |
| T203 | TMS5 |
| N330 | TMS7 |
| N331 | TMS7 |
| Y332 | TMS7 |
| F333 | TMS7 |
| F334 | TMS7 |
| Y335 | TMS7 |
| Y336 | TMS7 |
| N360 | TMS8 |
| F432 | TMS10 |
